# Network Pharmacology Combined with Experimental Validation to Investigate the Effects and Mechanisms of Aucubin on Aging-Related Muscle Atrophy

**DOI:** 10.3390/ijms26062626

**Published:** 2025-03-14

**Authors:** Wenan Li, Kaishu Deng, Mengyue Zhang, Yan Xu, Jingxi Zhang, Qingsheng Liang, Zhiyou Yang, Leigang Jin, Chuanyin Hu, Yun-Tao Zhao

**Affiliations:** 1 Guangdong Province Engineering Laboratory for Marine Biological Products, Guangdong Provincial Key Laboratory of Aquatic Product Processing and Safety, College of Food Science and Technology, Modern Biochemistry Experimental Center, Zhanjiang Municipal Key Laboratory of Marine Drugs and Nutrition for Brain Health, Guangdong Ocean University, Zhanjiang 524088, Chinadks377736843@163.com (K.D.); mengyuezhangzmy@163.com (M.Z.); 18670244665@163.com (Y.X.); 13531083526@163.com (J.Z.); lqs11073731@163.com (Q.L.); zyyang@gdou.edu.cn (Z.Y.); 2 State Key Laboratory of Pharmaceutical Biotechnology, Department of Medicine, The University of Hong Kong, Hong Kong SAR, China; jinlghk@hku.hk; 3 Department of Biology, Guangdong Medical University, Zhanjiang 524023, China

**Keywords:** Aucubin, muscle atrophy, oxidative stress, apoptosis, Sirt1/PGC-1α

## Abstract

Aucubin (AU) is one of the main components of the traditional Chinese medicine *Eucommia ulmoides* Oliv (EU). This study investigated the effects of AU on aging-related skeletal muscle atrophy in vitro and in vivo. The results of network pharmacology revealed the potential therapeutic effects of AU on muscle atrophy. In vitro, AU effectively attenuated D-gal-induced cellular damage, reduced the number of senescence-associated β-galactosidase (SA-β-Gal)-positive cells, down-regulated the expression levels of muscle atrophy-related proteins Atrogin-1 and MuRF1, and improved myotube differentiation, thereby mitigating myotube atrophy. Notably, AU was found to attenuate oxidative stress and apoptosis in skeletal muscle cells by reducing ROS production, regulating Cleaved caspase3 and BAX/Bcl-2 expression in apoptotic pathways, and enhancing Sirt1 and PGC-1α signaling pathways. In vivo studies demonstrated that AU treatment extended the average lifespan of *Caenorhabditis elegans* (*C. elegans)*, increased locomotor activity, improved body wall muscle mitochondrial content, and alleviated oxidative damage in *C. elegans*. These findings suggested that AU can ameliorate aging-related muscle atrophy and show significant potential in preventing and treating muscle atrophy.

## 1. Introduction

Muscle atrophy is a progressive and systemic skeletal muscle disease that presents itself as an accelerated loss of muscle mass and function. Its development is influenced by several factors, including chronic physical inactivity, nutritional deficiencies, chronic complications, and genetic susceptibility [1]. In addition, aging serves as a key contributor to this condition [2]. The global prevalence of aging-related muscle atrophy among adults aged 60 years and older is estimated to range from 10% to 27%, with severe cases accounting for 2% to 9% [3]. Individuals afflicted with muscle atrophy may experience difficulty maintaining an upright position due to the loss of muscle function. This can precipitate an elevated risk of falls and subsequent fractures, creating a safety hazard. Additionally, muscle atrophy can result in heart and lung failure, which can impact the function of other organs and may ultimately lead to death [4].

The pathogenesis of muscle atrophy is a complex process driven primarily by oxidative stress, abnormal mitochondrial function, chronic inflammation, apoptosis, and altered hormonal and growth factor signaling [5,6,7]. These processes are interconnected, with oxidative stress triggering mitochondrial dysfunction by impairing mitochondrial integrity, which in turn promotes apoptosis, while chronic inflammation amplifies protein degradation through a pro-catabolic state. Collectively, these factors contribute to the dysregulation of protein homeostasis, which represents the underlying mechanism of these conditions [8]. At present, the treatment of muscle atrophy focuses on encouraging physical activity, providing nutritional interventions, and pursuing other avenues of therapy [9]. However, these approaches have limitations, as physical activity may be challenging for frail individuals, and nutritional interventions often fail to fully reverse muscle loss in advanced cases. No pharmaceutical agents have been approved for the treatment of muscle atrophy [10]. With an increasingly aging population, there is a pressing need to develop more effective treatments for muscle atrophy.

Given the limitations of current treatments for muscle wasting, there is growing interest in exploring natural products as alternative therapeutic options. *Eucommia ulmoides* Oliv (EU), a traditional Chinese medicine, has the effects of nourishing the liver and kidneys, as well as strengthening tendons and bones [11]. Although EU has not been extensively tested in human clinical trials, studies have shown that herbal formulas containing EU can effectively improve muscle atrophy in rats, potentially through mechanisms involving enhanced antioxidant defense capacity and modulated inflammatory pathways [12]. As one of the main components of EU, Aucubin (AU) is a cyclic enol ether glycoside compound with a wide range of pharmacological activities and biological effects, including inhibition of apoptosis, antioxidation, anti-aging, anti-fibrosis, anti-cancer, liver protection, neuroprotection, and bone protection. Additionally, it effectively inhibits the endoplasmic reticulum stress response and attenuates inflammation [13,14,15,16]. AU’s multifaceted pharmacological profile, particularly its antioxidative and anti-apoptotic properties, suggests it may be a promising candidate for treating muscle atrophy.

To date, the effects and mechanisms of AU on muscle atrophy remain to be elucidated. This study aimed to explore whether AU can alleviate aging-related muscle atrophy and to determine the potential molecular mechanisms mediating its effects. Given AU’s multifaceted pharmacological profile, network pharmacology and molecular docking were employed to explore its potential in treating muscle atrophy, as these methods can effectively predict potential molecular targets and interactions. Additionally, the impact of AU on aging-related muscle atrophy and its underlying mechanisms were investigated in both in vivo and in vitro models.

## 2. Results

### 2.1. Network Pharmacology and Bioinformatics Analysis

**Figure 1:**
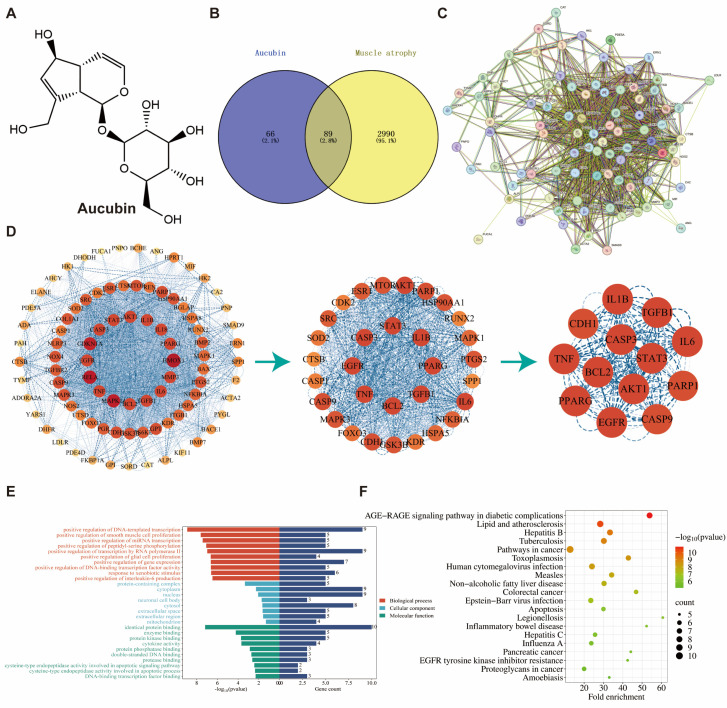
A preliminary screening and bioinformatics analysis of potential therapeutic gene targets for muscle atrophy. (**A**) Chemical structure of AU. (**B**) Venn diagrams of AU regulatory targets and muscle atrophy targets. (**C**) PPI network construction. (**D**) Core gene target screening. (**E**) Analysis of biological processes, cellular components, and molecular functions. (**F**) KEGG pathway analysis.

To investigate the potential molecular targets of AU for the treatment of muscle atrophy, we retrieved potential targets of AU and muscle atrophy-related disease targets from the databases in Section 4.2 of the Materials and Methods part. By identifying the intersection, we obtained 89 potential targets of AU for the treatment of muscle atrophy (Figure 1B). The protein–protein interaction (PPI) network was constructed using STRING. After removing the unconnected proteins, the network finally contained 88 nodes and 1179 edges, with an average node degree of 26.5, an average local clustering coefficient of 0.644, and a PPI-enriched *p*-value < 1.0 × 10^−16^ (Figure 1C). The data were analyzed using the CytoNCA plug-in with Cytoscape 3.9.1 software, and 13 core genes were identified through median screening (Figure 1D). These core genes were selected based on their high degree of connectivity and centrality within the network, reflecting their potential as key regulators. From a biological perspective, these genes are highly relevant to muscle wasting. For example, B-cell lymphoma 2 (Bcl-2), CASP3, and CASP9 are involved in regulating apoptosis, which impacts muscle cell survival, while IL6 and IL1B as pro-inflammatory cytokines promote muscle catabolism. These genes are associated with critical pathways, including protein degradation, inflammation, and mitochondrial dysfunction, which are central to the pathogenesis of muscle atrophy.

For bioinformatics analysis, we uploaded the 88 identified core targets into the database for Gene Ontology (GO) and Kyoto Encyclopedia of Genes and Genomes (KEGG) pathway enrichment analysis. This resulted in the identification of 202 biological processes (BP), 13 cellular components (CC), and 23 molecular function (MF) terms. We visualized the top 10 BP, CC, and MF terms (Figure 1E). In the BP category, positive regulation of DNA-triggered transcription, positive regulation of smooth muscle cell proliferation, and positive regulation of miRNA transcription were significantly enriched. In the CC category, protein-containing complexes, cytoplasm, nucleus, and mitochondria were notably enriched. In the MF category, significant enrichment was found for protein-binding, enzyme-binding, and cysteine-type endopeptidase activity involved in the apoptosis signaling pathway, as well as cysteine-type endopeptidase activity sites involved in the apoptosis process. Additionally, the KEGG pathway was used to predict the pathway of AU as a potential therapeutic target for muscle atrophy. A total of 101 statistically significant related pathways were identified, and the first 20 were selected for visualization (Figure 1F). These pathways included the AGE-RAGE pathway in diabetic complications, the cancer pathway, and apoptosis. Of these, apoptosis has been recognized as a key pathway in muscle atrophy [17,18,19].

### 2.2. Effects of AU on D-Galactose (D-Gal)-Induced C2C12 Cell Viability and Senescence

**Figure 2:**
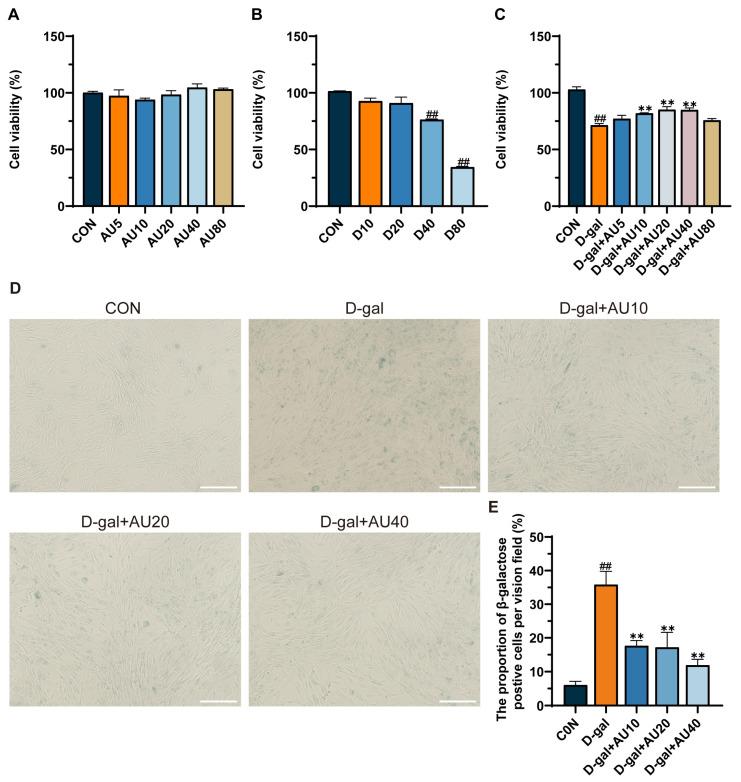
Effects of AU on D-gal-induced C2C12 cells. (**A**,**B**) Cell viability of AU and D-gal dose-dependently treated for 24 h. (**C**) The viability of D-gal-induced C2C12 cells was analyzed after 24 h-treatment with different concentrations of AU. (**D**) Representative images of SA-β-Gal staining in D-gal-induced C2C12 myoblasts treated with Phosphate-Buffered Saline (PBS) or AU for 24 h (bar = 100 μm). (**E**) Quantitative results of β-galactosidase staining. All data were expressed as mean ± SEM (*n* = 4). ^##^ *p* < 0.01 compared with control; ** *p* < 0.01 compared with D-gal group.

The results of the MTT assay showed that there was no significant difference in C2C12 cell viability when the concentration of AU was lower than 80 μM (Figure 2A). Next, C2C12 cells were treated with varying concentrations of D-gal (0, 10, 20, 40, and 80 mg/mL) for 24 h. As shown in Figure 2B, D-gal at 40 mg/mL significantly inhibited C2C12 cell proliferation (*p* < 0.01). Therefore, the 40 mg/mL D-gal concentration was selected to establish an in vitro model of muscle atrophy, which is consistent with findings reported by Nie et al. [20]. After AU treatment, it was found that AU at concentrations of 10, 20, and 40 μM significantly reversed the damage induced by D-gal in C2C12 cells (*p* < 0.01) (Figure 2C). Consequently, the optimal experimental concentrations were identified as 10, 20, and 40 μM AU.

Senescence-associated β-galactosidase (SA-β-Gal) staining is a commonly employed method for determining cellular senescence. The results demonstrated a significant increase in the number of SA-β-Gal-positive cells in the D-gal-induced group compared with the control group (*p* < 0.01). After treatment with AU, the numbers of SA-β-Gal-positive cells were significantly reduced (*p* < 0.01), indicating that AU significantly ameliorated D-gal-induced senescence of C2C12 (Figure 2D,E).

### 2.3. Effects of AU on D-Gal-Induced Myotube Diameter Atrophy

**Figure 3:**
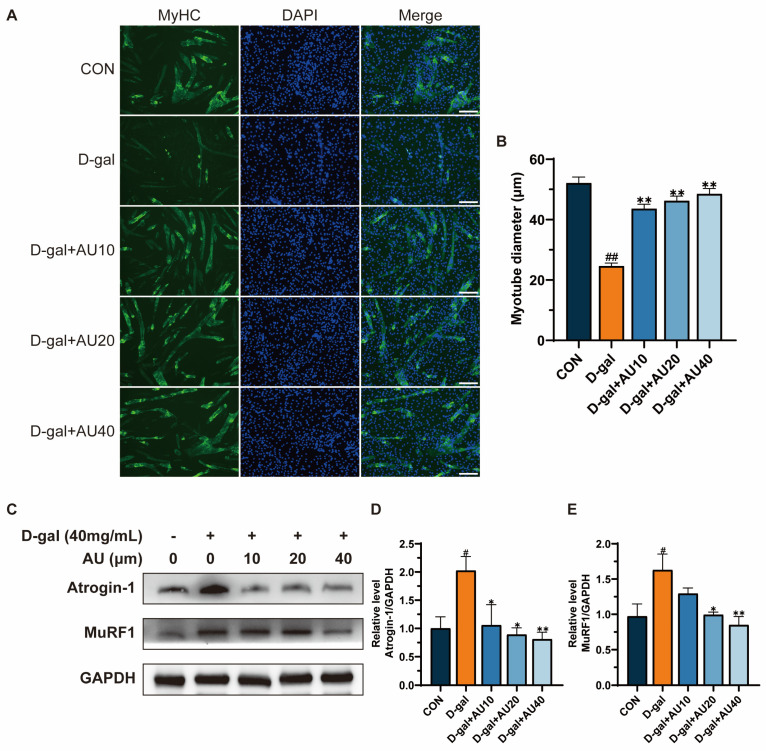
AU ameliorated D-gal-induced skeletal muscle atrophy. (**A**) Immunofluorescence staining of anti-MyHC antibody in mouse C2C12 myotubes (*n* = 40, bar = 100 μm). MyHC staining shows myotubes (green) and DAPI staining shows nuclei (blue). (**B**) Quantitative results of C2C12 myotube diameter. (**C**–**E**) Western blot analysis and quantification of Atrogin-1 and MuRF1 expression levels in C2C12 cells (*n* = 4). All data were expressed as mean ± SEM. ^##^ *p* < 0.01 and ^#^ *p* < 0.05 compared with control; ** *p* < 0.01 and * *p* < 0.05 compared with D-gal group.

Immunofluorescence staining of myosin heavy chain (MyHC) protein was used to assess myotube diameter. The results showed that the diameter of C2C12 myotubes in the D-gal-induced group was significantly reduced compared with that in the control group, suggesting that D-gal has a role in mimicking myotubular atrophy (Figure 3A,B). The diameter of myotubes in the AU treatment group was higher than that of the D-gal group, and showed an increase in the concentration gradient, suggesting that AU attenuated the myotubular atrophy induced by D-gal. 

The overexpression of muscle Atrophy F-box protein (Atrogin-1) and muscle RING-finger protein-1 (MuRF1), two key ubiquitin ligase E3s, is frequently considered an indicator of muscle atrophy [21]. The effects of AU on the expression levels of Atrogin-1 and MuRF1 were investigated through western blot analysis. As illustrated in Figure 3C–E, the expression levels of Atrogin-1 and MuRF1 were markedly elevated in the D-gal group in comparison to the control group (*p* < 0.05). Conversely, AU treatment demonstrated a dose-dependent reduction in the expression levels of Atrogin-1 and MuRF1.

### 2.4. Effects of AU on Oxidative Stress in D-Gal-Induced C2C12 Cells

**Figure 4:**
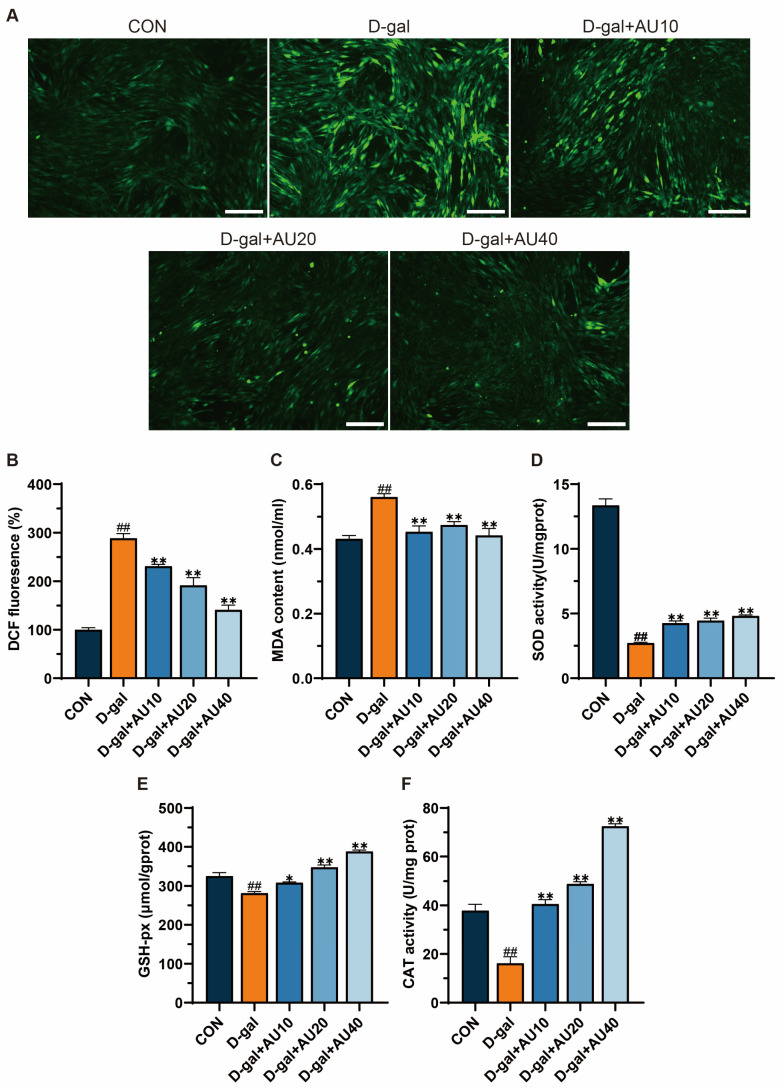
AU reduced D-gal-induced ROS production and improved redox homeostasis in C2C12. (**A**) Representative images of DCFH-DA-stained intracellular ROS in AU-treated C2C12 cells (bar = 100 µm). Levels of ROS (**B**), MDA (**C**), SOD (**D**), GSH-Px (**E**), and CAT (**F**) in the C2C12. All data were expressed as mean ± SEM (*n* = 4). ^##^ *p* < 0.01 compared with control; ** *p* < 0.01 and * *p* < 0.05 compared with D-gal group.

The overproduction of reactive oxygen species (ROS) and the subsequent oxidative stress that ensues are intimately linked to the process of senescent muscle atrophy [22]. Compared with the blank group, the D-gal group showed strong green fluorescence, indicating that D-gal could induce the increase of ROS level in C2C12 cells. The green fluorescence intensity decreased in a dose-dependent manner after AU treatment, indicating that AU could significantly reduce D-gal-induced ROS production (Figure 4A,B). Antioxidant enzymes such as catalase (CAT), superoxide dismutase (SOD), and glutathione peroxidase (GSH-Px) play crucial roles in combating oxidative stress and maintaining oxidative homeostasis. As shown in Figure 4C, malondialdehyde (MDA) levels in C2C12 cells were significantly elevated after 24 h of D-gal treatment compared with the control group. Following intervention with various concentrations of AU, intracellular MDA levels were significantly reduced. SOD, GSH-px, and CAT activities were dose-dependently increased in the AU-treated group compared with the D-gal-treated group, with the greatest increase observed in the high-concentration (40 μM) group (*p* < 0.01) (Figure 4D–F). In conclusion, these results suggest that AU exerts a protective effect against D-gal-induced oxidative stress.

### 2.5. Effects of AU on the Mitochondrial Membrane Potential (MMP) in D-Gal-Induced C2C12 Cells

**Figure 5:**
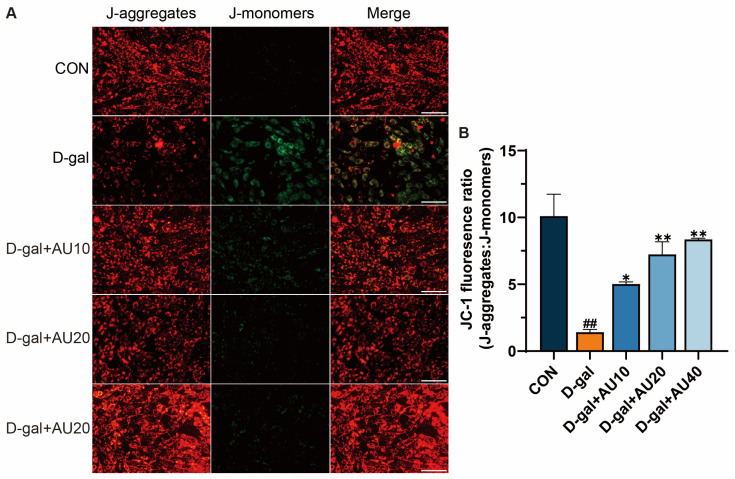
AU demonstrated efficacy in ameliorating D-gal-induced alterations in MMP. (**A**) Mitochondrial permeability was measured by JC-1 staining (J-aggregates show red fluorescence, and J-monomers show green fluorescence) (bar = 100 µm). (**B**) Quantitative analysis of J-aggregates/J-monomers. All data were expressed as mean ± SEM (*n* = 4). ^##^ *p* < 0.01 compared with control; ** *p* < 0.01 and * *p* < 0.05 compared with D-gal group.

The loss of MMP can lead to energy metabolism disorders and increased apoptosis in muscle cells [23]. It was shown that the J-aggregate/J-monomer ratio was significantly reduced in the D-gal-treated group compared with the control group. After AU treatment, the J-monomer/J-monomer ratio increased in a dose-dependent manner, with the most significant increase observed in the high-concentration (40 μM) group (Figure 5A,B). These results suggested that AU can improve the decrease of MMP induced by D-gal in C2C12 cells.

### 2.6. Effects of AU on D-Gal-Induced Apoptosis of C2C12 Cells

**Figure 6:**
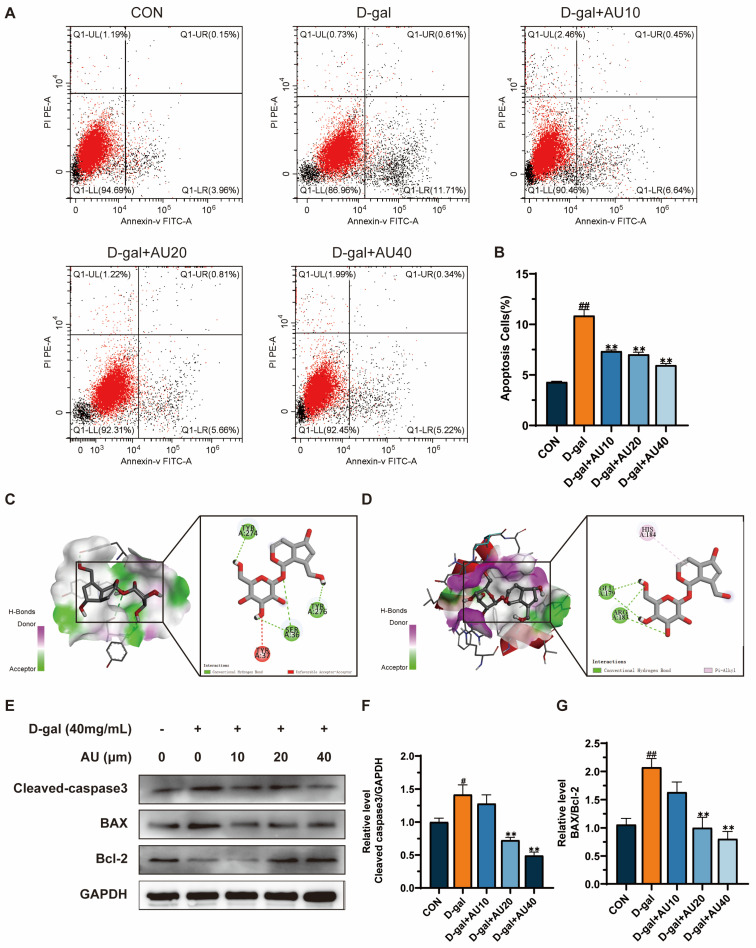
AU attenuated D-gal-induced apoptosis in C2C12 cells. (**A**) Flow cytometry was employed to assess the influence of AU on D-gal-induced apoptosis in C2C12 cells. (**B**) A statistical analysis of the apoptosis rate of cells in each group was performed. (*n* = 4). (**C**) Prediction of the three-dimensional structure of AU and caspase3 complexes by molecular docking with binding interactions of amino acid residues. (**D**) Binding interactions of the three-dimensional structure of the AU and Bcl-2 complex with amino acid residues predicted by molecular docking. (**E**) The impact of AU on the expression of Cleaved caspase3, BAX, and Bcl-2 proteins in D-gal-induced C2C12 cells was evaluated through western blotting. (**F**,**G**) A statistical analysis of protein expression in each group was conducted (*n* = 3). All data were expressed as mean ± SEM. ^##^ *p* < 0.01 and ^#^ *p* < 0.05 compared with control; ** *p* < 0.01 compared with D-gal group.

Network pharmacology results suggested that AU’s ability to alleviate muscle atrophy may be related to its regulation of apoptotic pathways. This study further investigated the targeted effects of AU on D-gal-induced muscle atrophy and elucidated its mechanism of action. First, the apoptosis rate of cells in each treatment group was examined by flow cytometry, and the results demonstrated that the proportion of cells undergoing early apoptosis was significantly higher in the D-gal group compared with the control group. The proportion of early apoptotic cells in the C2C12 cell line exhibited a gradual decline with increasing drug concentration following treatment with varying concentrations of AU, in comparison to the D-gal group (Figure 6A,B). These findings suggested that AU inhibits D-gal-induced apoptosis in C2C12 cells.

Furthermore, molecular docking techniques were employed to evaluate the potential interactions between AU and apoptosis-related core targets. Based on the results of the GO and the KEGG, apoptosis-related proteins such as caspase3 and Bcl-2 were selected as co-receptor proteins for molecular docking (Figure 6C,D). Molecular docking results demonstrated that AU could engage with the amino acid residues of target proteins through multiple interaction modes. Specifically, AU formed three conventional hydrogen bonds and one unfavorable acceptor–acceptor interaction with caspase3 (PDB ID 2J32), while interacting with Bcl-2 (PDB ID 8HTS) via two conventional hydrogen bonds and one pi–alkyl interaction. These docking scores suggested strong binding affinities, where hydrogen bonds conferred structural stability and binding specificity, the unfavorable acceptor–acceptor interaction introduced minimal repulsive effects, and the pi–alkyl interaction contributed hydrophobic stabilization [24]. Notably, the calculated binding energies for both targets were −6.5 kcal/mol, significantly lower than the threshold of −5 kcal/mol, further confirming that the combined contributions of these interactions ensured stable binding between AU and these core targets.

Western blot analysis further supported these findings, showing significantly elevated expression levels of Cleaved caspase3 and (BCL2-associated X protein) BAX/ Bcl-2 proteins in the D-gal group compared with the control group. Following treatment with 20 μM and 40 μM AU, the expression levels of Cleaved caspase3 and BAX/Bcl-2 proteins were significantly reduced in the D-gal group compared with the control group (Figure 6E–G). These findings were consistent with the results of network pharmacology and molecular docking, suggesting that AU may ameliorate apoptosis by modulating the expression of caspase3 and Bcl-2 proteins, thereby attenuating D-gal-induced muscle atrophy.

### 2.7. Effects of AU on D-Gal-Induced Sirt1/PGC1-α Signaling Pathway in C2C12 Cells

**Figure 7:**
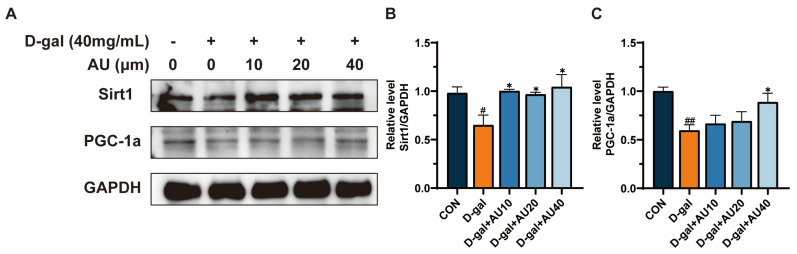
AU ameliorated D-gal-induced C2C12 aging-related muscle atrophy by modulating the Sirt1/PGC-1α signaling pathway. (**A**) The representative images of western blotting results. (**B**) Quantification of the protein expression of Sirt1. (**C**) Quantification of the protein expression of PGC-1α. All data were expressed as mean ± SEM (*n* = 4). ^##^ *p* < 0.01 and ^#^ *p* < 0.05 compared with control; * *p* < 0.05 compared with D-gal group.

The Sirt1/PGC-1α signaling pathway has been demonstrated to be closely associated with the process of aging-related muscle atrophy [25]. The protein expression levels of Sirt1 and PGC-1α in C2C12 cells were assessed by western blot. As shown in Figure 7A–C, the expression levels of Sirt1 and PGC-1α proteins in the D-gal group were significantly lower than those in the control group. Treatment with AU at concentrations of 10, 20, and 40 μM increased the expression levels of Sirt1 and PGC-1α proteins.

### 2.8. Effects of AU on the Lifespan and Behavior of the N2 Genotype of C. elegans

**Figure 8:**
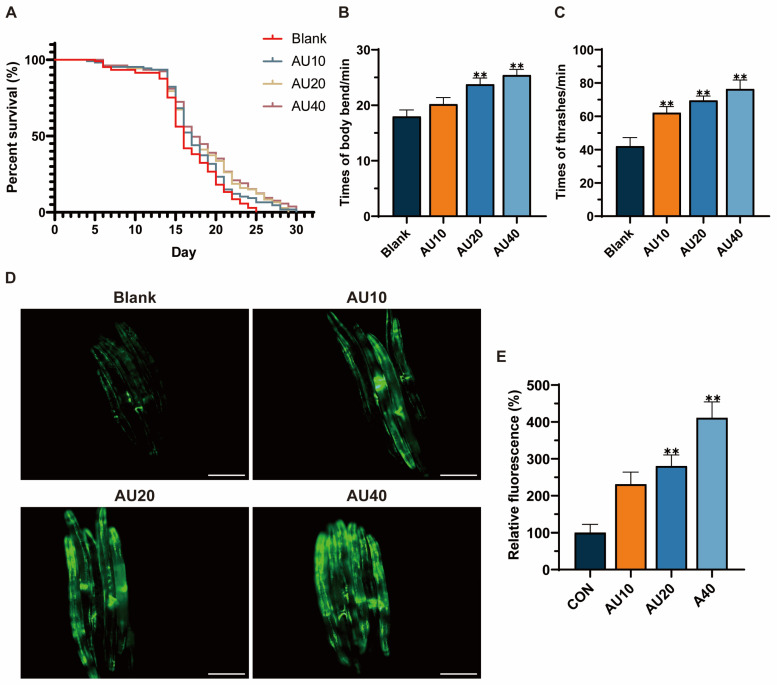
AU treatment prolonged lifespan, enhanced locomotion, and improved muscle health in the body wall of *C. elegans*. (**A**) Life curves of worms; (**B**) Times of body bends. (**C**) Times of thrashes. (**D**) Landmark images of mitochondrial contents and morphology of body wall muscle cells in SJ4103 worms (bar = 100 μm). (**E**) Quantitative analysis of mean fluorescence intensity (*n* = 30). All data were expressed as mean ± SEM. ** *p* < 0.01 compared with blank.

Lifespan extension has been identified as one of the most intuitive and convenient biological indicators of the ability to decelerate the aging process. The results demonstrated that the average lifespan of the control group was 15.56 days, compared with the blank group. The administration of AU treatments (20 and 40 μM) was observed to significantly prolong the lifespan of the worms by 10.20% and 12.92%, respectively (Figure 8A). Aging has been shown to have a detrimental impact on the musculature of worms, with movement capacity serving as an indicator of muscle health [26]. The findings revealed that the AU-treated group showed a significant improvement on head-bobbing and body-bending abilities, with the most significant enhancement observed in the high-concentration group (Figure 8B,C).

### 2.9. Effects of AU on Mitochondrial Content in the SJ4103 Genotype of C. elegans

SJ4103 is a wild-type strain that exhibits high levels of green fluorescent protein (GFP) expression in the mitochondria of body wall muscle cells. The intensity of GFP fluorescence is commonly used as a key marker to assess muscle health and atrophy [27]. To evaluate the effects of AU on aging-related muscle atrophy, worms were cultured with AU starting from the L4 stage and continuously exposed to the compound throughout their lifespan until reaching 8 days of age—a stage widely recognized as representative of aged *C. elegans* [28]. The results showed that AU treatment significantly increased GFP fluorescence expression in the body wall muscles of the worms compared with the control group (Figure 8D). Notably, the highest concentration (40 μM) produced the most substantial increase (Figure 8E). These findings suggested that AU may ameliorate muscle atrophy by improving mitochondrial content in worm body wall muscle during aging.

### 2.10. Effects of AU on Oxidative Stress in the N2 Genotype of C. elegans

**Figure 9:**
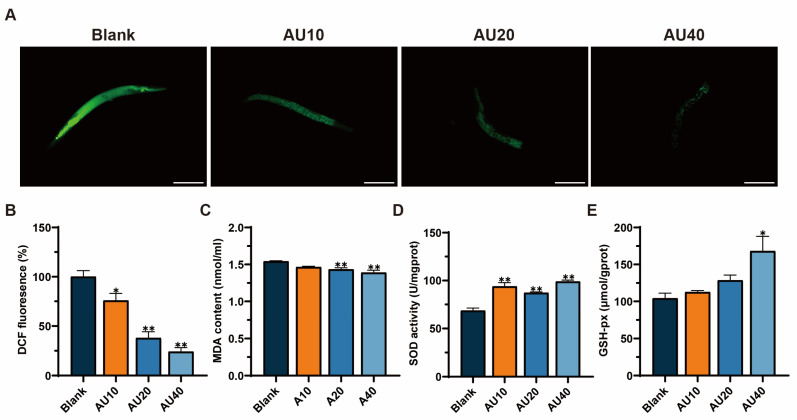
AU ameliorated the effects of ROS generation and oxidative stress during early aging in *C. elegans*. (**A**) Representative images of DCFH-DA-stained intracellular ROS in AU-treated worms (bar = 100 µm). Levels of ROS (**B**), MDA (**C**), SOD (**D**), and GSH-Px (**E**) in the *C. elegans*. All data were expressed as mean ± SEM (*n* = 30). ** *p* < 0.01 and * *p* < 0.05 compared with blank.

Oxidative stress represents a significant contributing factor to the process of nematode aging. The levels of ROS and MDA, as well as the activities of SOD and GSH-Px in *C. elegans* were measured. The results showed that after treatment with AU for 3 days, ROS and MDA levels in the worms decreased in a dose-dependent manner compared with the blank group (Figure 9A–C). As shown in Figure 9D,E, AU treatment could significantly increase the activities of SOD and GSH-px. These findings suggested that AU can reduce the level of oxidative stress in *C. elegan*s during early aging.

## 3. Discussion

Natural products with distinct biological properties show significant potential for treating muscle atrophy [1]. In this study, network pharmacology was used to study the protective effect and potential mechanism of AU on muscle atrophy. In vitro results showed that D-gal-induced aging-related markers, including oxidative stress and apoptosis, in C2C12 and AU intervention could reverse these changes. Additionally, in vivo experiments revealed that AU intervention promoted longevity, improved locomotor function, and increased mitochondrial content in the body wall muscle of *C. elegans*.

The regulation of the balance between protein degradation and synthesis is a critical strategy for mitigating muscle atrophy. Abnormal activation of the ubiquitin–proteasome pathway leads to the accelerated degradation of muscle proteins [29]. MyHC is a key protein for muscle contraction, and Wu et al. demonstrated that D-gal treatment inhibited the expression of MyHC, which led to muscle atrophy [30]. MuRF1 and Atrogin-1 can promote the degradation of muscle structural and functional proteins through ubiquitination tagging [31]. In our experiments, AU treatment significantly inhibited the overexpression of MuRF1 and Atrogin-1 and reversed the D-gal-induced reduction in MyHC expression, thereby ameliorating D-gal-induced muscle atrophy.

Oxidative stress has been identified as a pivotal factor in the progression of muscle atrophy [32,33]. In a model of D-gal-induced skeletal muscle atrophy, elevated levels of oxidative stress promote ROS production and inhibit intracellular antioxidant enzyme activities, which in turn exacerbate muscle cell damage [34]. A similar phenomenon has been observed in senescent conditions, where the continuous accumulation of ROS in *C. elegans* has been shown to result in muscle cell damage and, consequently, accelerate muscle atrophy [26]. These findings are consistent with our study, which showed that D-gal treatment significantly elevated ROS and MDA levels while reducing antioxidant enzyme activities in C2C12 cells compared to the control group. AU intervention reversed these oxidative stress-induced changes, likely by enhancing antioxidant defenses, such as SOD activity, and mitigating cellular damage. This is consistent with findings from studies on other natural compounds, such as *Lycium barbarum* cortex, which similarly reduces ROS and enhances antioxidant capacity in muscle atrophy models to ameliorate damage in C2C12 cells [35]. Similarly, in the *C. elegans* model, AU treatment for three days reduced oxidative stress during aging by reversing these alterations, potentially improving locomotion through the protection of muscle function from oxidative damage. Increased ROS production has been shown to reduce MMP in skeletal muscle cells, which can compromise the structural integrity of the inner mitochondrial membrane and exacerbate muscle atrophy. In this study, we observed that D-gal treatment resulted in a decline in C2C12 MMP, a finding that aligns with the study conducted by Wang et al. [34]. Notably, AU administration effectively counteracted this decline, suggesting its potential to enhance MMP by reducing ROS-induced mitochondrial damage, which may indirectly support increased mitochondrial content in the *C. elegans*, although further studies are needed to confirm this effect.

Muscle atrophy is frequently accompanied by apoptosis [36,37,38]. Previous studies have demonstrated that D-gal treatment hinders myogenic differentiation and leads to apoptosis [25,39]. This finding is consistent with our observations, which demonstrated a significant increase in early apoptosis in the D-gal-induced group compared with the control group. However, apoptosis was significantly inhibited following AU intervention. Bcl-2 family proteins (including anti-apoptotic Bcl-2 and pro-apoptotic BAX) are key regulators of apoptosis [40,41]. Research has shown that upregulation of BAX and downregulation of Bcl-2 during muscle atrophy alter the permeability of the outer mitochondrial membrane, triggering the release of pro-apoptotic factors such as cytochrome C and activating the caspase cascade [42,43]. The activation of Cleaved caspase3 (the final executioner of apoptosis) signals the conclusion of cell death and initiates apoptosis in muscle cells [44]. In this study, AU intervention resulted in decreased BAX expression and increased Bcl-2 levels, thereby inhibiting the mitochondria-dependent apoptotic pathway. This is consistent with reports on guava leaf extract, which also ameliorates muscle atrophy by modulating the expression levels of apoptosis-related proteins such as BAX and Bcl-2, although it additionally explores effects potentially mediated by autophagy [45]. Molecular docking analysis showed that AU may stabilize its anti-apoptotic conformation through pi–alkyl interaction and hydrogen bond with Bcl-2, thereby enhancing its anti-apoptotic effects. Furthermore, AU intervention led to a significant reduction in Cleaved caspase3 expression, indicating that AU reduces apoptosis in muscle cells by preventing the activation of the caspase cascade. Molecular docking simulations suggested that these effects may arise from the formation of stable binding between AU and the active site of caspase3 via hydrogen bonds and unfavorable receptor–receptor interactions, thereby inhibiting its activation.

**Figure 10:**
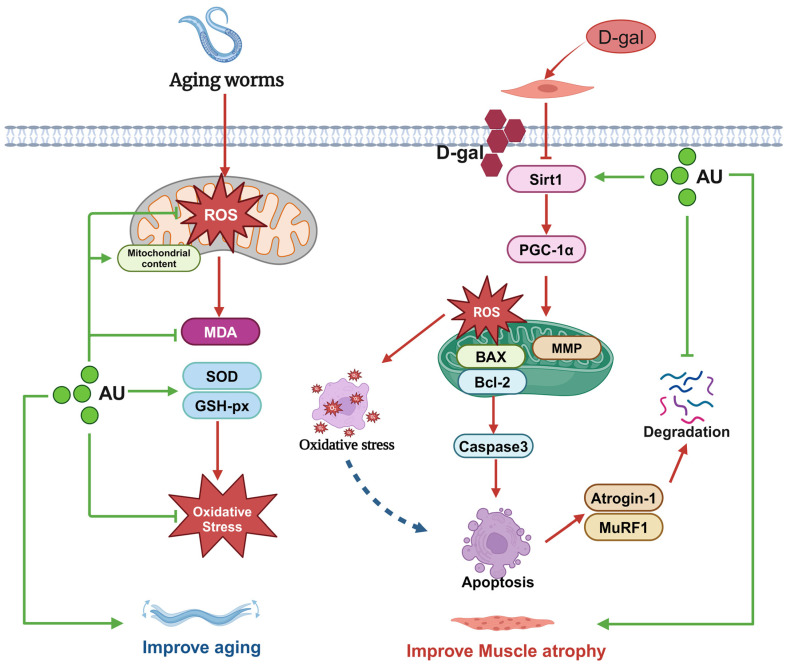
Schematic representation of the possible mechanisms of the anti-aging muscle atrophy effects of AU.

The Sirt1/PGC-1α pathway plays a crucial role in the regulation of muscle homeostasis [23]. Sirt1, a NAD^+^-dependent deacetylase, mitigates oxidative stress by deacetylating and activating key antioxidant enzymes [46]. Moreover, Sirt1 regulates apoptotic processes by deacetylating transcription factors and suppressing the expression of pro-apoptotic proteins, thereby inhibiting apoptosis [47]. PGC-1α, a central regulator of mitochondrial function, acts downstream of Sirt1 to promote cellular homeostasis by enhancing mitochondrial biogenesis and oxidative metabolism [48]. Multiple studies have shown that activation of the SIRT1/PGC-1α pathway can inhibit the expression of Atrogin-1 and MuRF1, thereby reducing muscle protein degradation [49,50]. In this study, we examined the protein expression levels of Sirt1 and PGC-1α. The results showed that D-gal inhibited the expression of the Sirt 1/PGC-1α pathway and AU reversed these effects. These findings suggested that AU may ameliorate muscle atrophy by activating the Sirt1/PGC-1α signaling pathway, which modulates oxidative stress and apoptosis to reduce the expression of Atrogin-1 and MuRF1 (Figure 10). In addition, Sirt1 and PGC-1α also interact with a variety of other muscle metabolism-related pathways, such as the AMPK pathway [23], involved in the regulation of glucose and lipid metabolism [51], and the calcium signaling pathway [52]. Therefore, the effects of AU on muscle atrophy may not be completely dependent on the Sirt1/PGC-1α pathway but may also play a role by affecting these other pathways, which needs to be further investigated in future studies.

Collectively, our findings demonstrated that AU intervention consistently mitigates oxidative stress and related cellular damage across both in vivo *C. elegans* and in vitro C2C12 skeletal muscle cell models, suggesting its potential as a therapeutic agent to counteract aging-related muscle atrophy by enhancing antioxidant defenses and preserving mitochondrial integrity. Furthermore, unlike many natural compounds that regulate apoptosis primarily through indirect pathways, AU directly interacts with Bcl-2 and caspase3, modulating their activity to restore apoptosis balance, suggesting that AU may exert a more precise anti-apoptotic effect. Our study provides a foundation for developing AU-based drugs or optimizing its structure to enhance its efficacy in ameliorating muscle atrophy.

However, our study has certain limitations. In our in vitro experiments, we used healthy C2C12 cells to study D-gal-induced aging and oxidative stress. Of note, this model cannot fully recapitulate the aging-associated muscle atrophy observed in vivo. In addition, *C. elegans* also cannot fully replicate mammalian physiology; therefore, further studies (e.g., mice) are needed to clarify the impact of AU on muscle atrophy during aging.

## 4. Materials and Methods

### 4.1. Reagents

C2C12 myoblasts were purchased from the Cell Bank of the Chinese Academy of Sciences (Shanghai, China). D-gal was purchased from Sigma (St. Louis, MO, USA). AU (≥98.0%, purity) was obtained from Shanghai Yuan-ye Biotechnology Co. (Shanghai, China). Fetal bovine serum (FBS) was supplied by Zhejiang Tianhang Biotechnology Co., Ltd. (Hangzhou, China). HS, Dulbecco’s Modified Eagle Medium (DMEM), penicillin, and trypsin were purchased from Gibco (Grand Island, NE, USA). The ROS assay kit and bicinchoninic acid (BCA) assay kit were obtained from Beyotime Biotechnology (Shanghai, China). The SA-β-Gal Staining Kit was purchased from Beijing Solarbio Science & Technology Co., Ltd. (Beijing, China). The MDA kit and SOD kit were purchased from Suzhou Grace Biotechnology (Nanjing, China), while GSH-Px and CAT were obtained from the Nanjing Jiancheng Bioengineering Institute (Nanjing, China). Antibodies against glyceraldehyde-3-phosphate dehydrogenase (GAPDH) were purchased from Cell Signaling Technology (Danvers, MA, USA). Polyclonal antibodies against MyHC, BAX, Bcl-2, and Cleaved caspase3 were purchased from Santa Cruz Biotechnology (Dallas, TX, USA). Polyclonal antibodies against Sirtuin 1 (Sirt1), Atrogin-1, peroxisome proliferator-activated receptor gamma coactivator 1-alpha (PGC-1α), and MuRF1 were obtained from Abcam (Cambridge, UK). All other reagents were of analytical grade.

### 4.2. Identification of Targets

Canonical Structure Data File (SDF) structure of AU was searched using PubChem (https://pubchem.ncbi.nlm.nih.gov/, accessed on 16 October 2024). By uploading the canonical SDF structure to Swiss Target Prediction database (http://www.swisstargetprediction.ch/, accessed on 16 October 2024), CTD (https://ctdbase.org/, accessed on 16 October 2024), Pharmmapper (https://lilab-ecust.cn/pharmmapper/index.html/, accessed on 17 October 2024), and TCMSP (https://www.tcmsp-e.com/load_intro.php?id=43, accessed on 17 October 2024) [53] databases to predict the potential target genes of AU. Disease targets associated with muscle atrophy were identified using GeneCards (https://www.genecards.org/, accessed on 17 October 2024) [54], and OMIM (https://www.omim.org/, accessed on 17 October 2024) databases [55]. The UniProt database (https://www.uniprot.org/, accessed on 17 October 2024) was utilized to standardize the target names and the transformed gene names [56]. Finally, the intersections were identified and extracted through the Venny 2.1 website (https://bioinfogp.cnb.csic.es/tools/venny/, accessed on 17 October 2024) as the potential targets for AU treatment of muscle atrophy.

### 4.3. PPI Analysis

The common targets of AU and muscle atrophy were uploaded into an online tool string database with a confidence score greater than 0.40. A “drug-disease-target-pathway” network diagram was constructed using Cytoscape (version 3.9.1) [57].

### 4.4. Establishment of GO and KEGG Enrichment Network Graphs

GO and KEGG Pathway enrichment analysis of AU on improving muscle atrophy were completed through the David Database (https://David.ncifcrf.gov/, accessed on 18 October 2024) [58]. Pathway and GO results with *p*-values less than 0.05 were selected. Furthermore, the online mapping tools described above were employed to generate GO and KEGG enrichment plots and Venn diagrams [59].

### 4.5. Molecular Docking

To assess the binding energy and interaction modes between AU and its targets, AutoDock 1.5.7 was used to perform flexible docking of ligands to proteins and to identify potential binding cavities [60]. The X-ray crystal structures of caspase3 and Bcl-2 proteins were downloaded from the Protein Data Bank. The docking scores and rankings of AUs with caspase3 and Bcl-2 protein receptors were generated using the Vina tool to reflect the binding affinity of AUs to the receptors. Discovery Studio 2021 Client (BIOVIA, San Diego, CA, USA) was used for visualization and analysis of molecular docking in this study [24].

### 4.6. Cell Culture and Treatment

C2C12 myoblasts were cultured in DMEM medium containing 10% fetal bovine serum and 1% penicillin/streptomycin (P/S) in a humidified incubator at 37 °C with 5% CO_2_. To induce the fusion of myoblasts into myotubes, 2% HS was employed instead of fetal bovine serum for 5–6 days.

To determine the effects of D-gal and AU in differentiated C2C12 cells. C2C12 cells were randomly assigned to control, D-gal, and AU treatment groups. Cells in the control group were incubated in 2% HS for 24 h. The D-gal group was treated with 2% HS containing D-gal (40 mg/mL) for 24 h. The AU group was treated with 2% HS DMEM solution containing D-gal (40 mg/mL) and varying concentrations of AU (10, 20, and 40 μM) for 24 h.

### 4.7. Cell Viability Assay

MTT assays were used to evaluate the impact of AU and D-gal on C2C12 cell proliferation. C2C12 cells were inoculated into 96-well plates at a density of 2 × 10^5^ cells/well and cultured for 24 h. Following this, the cells were rinsed twice with PBS, and untreated wells were maintained as absolute controls, while other wells were exposed to different concentrations of D-gal or AU for the indicated times. Subsequently, 150 μL of MTT solution (0.5 mg/mL) was added to each well and incubated for 4 h. The supernatant was discarded and 200 μL of Dimethyl Sulfoxide (DMSO) was added to dissolve the formazan. The absorbance was measured at 490 nm using an enzyme reader.

### 4.8. SA-β-Gal Staining

SA-β-Gal staining was performed using the SA-β-Gal staining kit according to the manufacturer’s instructions. The treated cells were washed with PBS and fixed with β-Gal staining fixative for 15 min at room temperature. Washed three times with PBS, 1 mL staining solution was added to each well and cultured in a 37 °C dry incubator (No CO_2_). The number of positive cells was quantified through microscopic observation under an inverted phase-contrast microscope.

### 4.9. Measurement of Myotube Diameters

The MyHC staining experiment was carried out according to the previous article with some adjustments [61]. The differentiated C2C12 cells were treated with D-gal or AU for 24 h. Following the removal of the medium and two washes with PBS, the C2C12 cells were fixed in 4% paraformaldehyde for 15 min at room temperature. The cells were washed thrice with PBS containing 0.1% Triton X-100. Following this, the cells were incubated with Bovine Serum Albumin for 2 h. The cells were incubated with anti-MyHC antibodies overnight at 4 °C. After being washed three times, the fluorescent secondary antibody was incubated for 1 h at room temperature. Afterward, the cells were stained with 4′,6-diamidino-2-phenylindole for 5 min. The myotubes were imaged with a fluorescence microscope. Myotube diameters in the field of view were analyzed using Image J software (version 1.53), the diameters of a total of 40 myotubes from at least 5 random fields of view in each group were measured.

### 4.10. Determination of Antioxidant Activity in C2C12 Cells

Cells cultured in 6-well plates were treated with 1 mL of DCFH-DA probe at a concentration of 10 μM for 30 min in the dark at 37 °C. The cells were washed twice with PBS and imaged using an inverted fluorescence microscope (DMI4000B, Leica, Wetzlar, Germany). The intensity of green fluorescence in the field of view was quantified using Image J software.

After collecting cells from the various treatment groups, the cells were disrupted using ultrasound, and the resulting supernatant was centrifuged in preparation for subsequent assays. The concentrations of MDA, SOD, GSH-Px, and CAT were quantified in C2C12 cells using the methods described in the accompanying kit. The protein content was determined using a BCA assay kit.

### 4.11. MMP Assay

MMP was assessed using a JC-1 assay kit. Cells from the various treatment groups were incubated in DMEM containing the JC-1 working solution at 37 °C for 20 min and washed twice with PBS solution. JC-1 aggregates (red fluorescence) and JC-1 monomers (green fluorescence) were imaged with an inverted fluorescence microscope (DMI4000B, Leica, Germany), and the fluorescence intensity within the field of view was quantified using Image J software.

### 4.12. Apoptosis Analysis by Flow Cytometry

Cells from the different administration groups were treated according to the instructions provided in the Annexin V-FITC/PI Double Staining Kit. Apoptosis in C2C12 cells was detected using flow cytometry, and the data were analyzed using CytExpert software (version 2.5).

### 4.13. Western Blot

Cells were collected and the total proteins were extracted using RIPA lysis buffer. The protein concentration in the samples was determined using a BCA assay kit. The proteins were separated by SDS-PAGE and transferred to a PVDF membrane in moderate amounts. After blocking at room temperature for one hour with no-fat milk, the membranes were incubated overnight at 4 °C with primary antibodies specific for GAPDH, Atrogin-1, Murf1, Sirt1, PGC-1, Cleaved caspase, Bcl-2, and BAX. The membranes were washed with TBST and incubated with the secondary antibody for one hour. Protein bands were detected using an ECL kit in a chemiluminescence system, and the bands were analyzed using Image J software to quantify their intensity. The GAPDH protein was used as an internal reference standard.

### 4.14. C. elegans Strains and Culture

The *C. elegans* strains N2 (wild-type), SJ4103 [zcIs14] and *Escherichia coli* (*E. coli*) OP50 strain were obtained from the Caenorhabditis Genetics Center (CGC), University of Minnesota (Minneapolis, MN, USA). *C. elegans* were cultured under the following conditions: the worms were cultivated on NGM agar plates at 20 °C. The worms were synchronized using a lysis solution of 1.1 mL of sterile water, 0.5 mL of NaOH (2 M), and 0.4 mL of NaClO (100%) solution. Following synchronization, the worms were cultured for 48 h to reach the L4 stage and then divided into different treatment groups to prepare for the experiment.

### 4.15. Lifespan Assay

AU was dissolved in PBS, mixed with inactivated OP50, and distributed uniformly on NGM plates. The synchronized N2 worms at L4 stage were cultured on AU plates (10, 20, 40 μM) or blank plates. A total of 34 worms were placed on each plate, with three plates assigned to each group. The transfer day was designated as day 0, and worms were transferred to fresh extracts or blank plates every other day until all died. The survival of the worms were recorded daily by the tactile excitation method, while abnormal worms were excluded from the analysis. Statistical analyses were performed using GraphPad software.

### 4.16. Motility

Motility assays were performed as previously described with slight modifications [27]. For the N2 strain, the L4 stage worms were transferred to NGM plates containing varying concentrations of AU. The plates were incubated continuously for 10 days to simulate natural senescence. Worms were placed on microscope slides and a drop of M9 buffer was added. The sum of the number of body bends and head-bobbing in 10 s was counted and multiplied by 6 to calculate the rate of movement per minute. Three measurements were taken for each worm to obtain an average rate.

### 4.17. Fluorescence Measurements of Mitochondria

SJ4103 worms were treated in Petri dishes with or without AU for 8 days, then collected and washed three times with M9 buffer [26]. The worms were subsequently placed on 2% agarose pads containing levamisole solution as an anesthetic on glass microscope slides and observed using a fluorescence microscope (DMI4000B, Leica, Germany). Quantitative analysis of fluorescence was performed using Image J (*n* = 30).

### 4.18. Oxidative Stress Levels of C. elegans

The determination was conducted following the previously described methods, with slight modifications [62]. The levels of ROS in worms were assessed using 2′,7′-dichlorofluorescein diacetate (DCFH-DA). The N2 strain of *C. elegans* at the L4 stage were treated with different concentrations of AU for three days. Worms were incubated with 10 μM DCFH-DA at 37 °C for 20 min, followed by imaging with a fluorescence microscope (DMI4000B, Leica, Wetzlar, Germany). The fluorescence intensity in the field of view was quantified using Image J software.

The collected worms were crushed, centrifuged, and the supernatant was collected and assayed for MDA, SOD, and GSH-Px according to the manufacturer’s protocol.

### 4.19. Statistical Analysis

Survival analyses were performed by the Kaplan–Meier method. All results were expressed as the mean ± standard error of the mean (SEM) of at least three independent experiments. Marked differences (*p* < 0.05) between different experimental groups were determined using one-way ANOVA followed by Duncan’s multiple-range post-hoc analysis. The statistical analysis was conducted using GraphPad Prism 8 software.

## 5. Conclusions

In conclusion, the findings suggested that AU exerted protective effects against muscle atrophy both in vivo and in vitro. AU demonstrated the ability to reduce oxidative stress and inhibit apoptosis by activating the Sirt1/PGC-1α signaling pathway. This process effectively reversed D-gal-induced muscle atrophy in C2C12 senescent cells. Furthermore, AU improved aging and muscle health in *C. elegans* by reducing oxidative stress and enhancing mitochondrial content in the body wall. These pathways undoubtedly play a positive role in improving muscle atrophy, but future research should explore other potential mechanisms to fully determine the scope of its treatment, thus providing new possibilities for the treatment of aging-related muscle atrophy.

## Data Availability

The data presented in this study are available on request from the corresponding author.

## Abbreviations

The following abbreviations are used in this manuscript:

Atrogin-1	Muscle Atrophy F-box protein
AU	Aucubin
BAX	BCL2-associated X protein
BCA	Bicinchoninic acid
Bcl-2	B-cell lymphoma 2
BP	Biological process
*C. elegans*	*Caenorhabditis elegans*
CAT	Catalase
CC	Cellular component
CGC	Caenorhabditis Genetics Center
D-gal	D-galactose
DCFH-DA	2′,7′-dichlorofluorescein diacetate
DMEM	Dulbecco’s Modified Eagle Medium
DMSO	Dimethyl Sulfoxide
*E. coli*	*Escherichia coli*
EU	*Eucommia ulmoides* Oliv
FBS	Fetal bovine serum
GAPDH	Glyceraldehyde-3-phosphate dehydrogenase
GFP	Green fluorescent protein
GO	Gene Ontology
GSH-Px	Glutathione peroxidase
HS	Horse serum
KEGG	Kyoto Encyclopedia of Genes and Genomes
MDA	Malondialdehyde
MF	Molecular function
MMP	Mitochondrial membrane potential
MuRF1	Muscle RING-finger protein-1
MyHC	Myosin heavy chain
PBS	Phosphate-Buffered Saline
PGC-1α	Peroxisome proliferator-activated receptor gamma coactivator 1-alpha
PPI	Protein-protein interaction
ROS	Reactive oxygen species
SA-β-Gal	Senescence-associated β-galactosidase
SDF	Canonical Structure Data File
SEM	Standard Error of the Mean
Sirt1	Sirtuin 1
SOD	Superoxide dismutase
